# Role of Toll-Like Receptor Signaling in the Pathogenesis of Graft-versus-Host Diseases

**DOI:** 10.3390/ijms17081288

**Published:** 2016-08-11

**Authors:** Sanfang Tu, Danli Zhong, Weixin Xie, Wenfa Huang, Yangyang Jiang, Yuhua Li

**Affiliations:** 1 Department of Haematology, Zhujiang Hospital, Southern Medical University, Guangzhou 510282, China; doctortutu88@gmail.com (S.T.); jyy880202@gmail.com (Y.J.); 2 Second Clinical Medical College, Zhujiang Hospital, Southern Medical University, Guangzhou 510282, China; drzhong0306@gmail.com (D.Z.); xwx1994519@gmail.com (W.X.); dsrhwf@gmail.com (W.H.)

**Keywords:** TLRs, GVHD, PAMPs, HSCT

## Abstract

Graft-versus-host disease (GVHD) and infection are major complications after allogeneic hematopoietic stem cell transplantation (allo-HSCT) and the leading causes of morbidity and mortality in HSCT patients. Recent work has demonstrated that the two complications are interdependent. GVHD occurs when allo-reactive donor T lymphocytes are activated by major histocompatibility antigens or minor histocompatibility antigens on host antigen-presenting cells (APCs), with the eventual attack of recipient tissues or organs. Activation of APCs is important for the priming of GVHD and is mediated by innate immune signaling pathways. Current evidence indicates that intestinal microbes and innate pattern-recognition receptors (PRRs) on host APCs, including both Toll-like receptors (TLRs) and nucleotide oligomerization domain (NOD)-like receptors (NLRs), are involved in the pathogenesis of GVHD. Patients undergoing chemotherapy and/or total body irradiation before allo-HSCT are susceptible to aggravated gastrointestinal epithelial cell damage and the subsequent translocation of bacterial components, followed by the release of endogenous dangerous molecules, termed pathogen-associated molecular patterns (PAMPs), which then activate the PRRs on host APCs to trigger local or systemic inflammatory responses that modulate T cell allo-reactivity against host tissues, which is equivalent to GVHD. In other words, infection can, to some extent, accelerate the progression of GVHD. Therefore, the intestinal flora’s PAMPs can interact with TLRs to activate and mature APCs, subsequently activate donor T cells with the release of pro-inflammatory cytokines, and eventually, induce GVHD. In the present article, we summarize the current perspectives on the understanding of different TLR signaling pathways and their involvement in the occurrence of GVHD.

Allogeneic hematopoietic stem cell transplantation (allo-HSCT) is currently the only radical therapy for a wide range of benign and aggressive hematopoietic malignancies. However, the approach has limited efficacy due to lethal complications such as graft-versus-host disease (GVHD) and infection, the leading causes of morbidity and mortality in HSCT patients [1]. GVHD is considered as an accentuated inflammatory response triggered by donor T cells, and the immune system plays an essential role in the pathogenesis of GVHD [2]. Increasing evidence shows that the innate immune system bridges the association between infection and GVHD [3]. Pattern-recognition receptors (PRRs) play a key role in the innate immune system by sensing pathogen-associated molecular patterns (PAMPs) and danger-associated molecular patterns (DAMPs), and subsequently initiating immune responses. Among the innate immune receptors, PRRs, two families have been acknowledged as key molecules in the pathogenesis of GVHD: membrane-bound receptors termed Toll-like receptors (TLRs) and cytoplasmic nucleotide oligomerization domain (NOD)-like receptors (NLRs) which are localized in the cytoplasmatic compartment [4,5]. Most PRRs are present on host and donor antigen-presenting cells (APCs), including dendritic cell (DC) subsets, neutrophils, monocytes, macrophages and natural killer cells, while some are distributed on epithelia and organ parenchymal cells. Generally, PAMPs are small molecular motifs conserved within a class of microbes and are considered as exogenous microbial molecules including lipopolysaccharide (LPS), RNA, flagellen, etc., while DAMPs are associated with endogenous cell components that are released during cell damage or death, including high mobility group box 1, s100 proteins, elastase inhibitors (endogenous proteases inhibitors), defensins, cathelicidins, heat shock proteins, heparan sulfate proteoglycans, etc. [6,7]. Patients undergoing conditioning regimens including intensive chemotherapy and/or total-body irradiation (TBI) are susceptible to aggravated intestinal tissue damage caused by microflora [8,9], with subsequent translocation of gut microbes and microbial products, especially PAMPs, and the release of endogenous DAMPs. It leads to the production of pro-inflammatory cytokines and the recruitment of allo-reactive donor T cells to target tissues or organs, including the skin, gastrointestinal tract and liver, which are under long-term exposure to PAMPs [10]. In summary, infection can induce the occurrence and progression of GVHD, which is associated with the innate immune system. The innate immune system enhances adaptive immunity by activating donor T cells to produce massive cytokines, and then triggers or intensifies GVHD. Thus, it is essential to understand the role of innate immunity in the occurrence of GVHD for new approaches for prevention and therapies of GVHD. In the present article, we summarize the current research supporting the involvement of different TLR signaling pathways in the pathogenesis of GVHD.

## 1. Introduction to Toll-Like Receptors (TLRs)

TLRs constitute a family of transmembrane innate PRRs that are broadly expressed by both non-hematopoietic and hematopoietic cells [11]. In mammals, TLRs are present on various immune cells, including natural killer cells, DC subsets, monocytes, macrophages, neutrophils, T lymphocytes and B lymphocytes. Some non-hematopoietic epithelial, endothelial and organ parenchymal cells also express these molecules. TLRs, as evolutionarily conserved molecules, were first described in vertebrates as proteins homologous to the insect molecule *Toll*, which could activate the secretion of antimicrobial peptides in *Drosophila melanogaster* [12]. Thirteen TLRs (TLR1 to TLR13) have been identified in both humans and mice, and various equivalent forms of these receptors have been discovered in other mammalian species. However, equivalent forms of certain TLRs found in humans are not present in all mammals [13]. For example, it has been found that mice express TLR11, TLR12 and TLR13, but none of them is represented in humans. Other non-mammalian species, for instance the Takifugu pufferfish, have been found to express TLR14 which cannot be found in mammals [13]. TLRs are now identified as key molecules that alert the immune system to the presence of microbial infections through signal transduction. TLR1 (functioning in TLR1/2 heterodimers), TLR2 (receptor for glycolipids, lipoteichoic acid, bacterial lipoprotein and components of mycobacterial walls), TLR4 (receptor for LPS, respiratory syncytial virus F protein, glycoinositolphospholipids, heat shock proteins and fibrinogen), TLR5 (receptor for bacterial flagellar protein), TLR6 (functioning in TLR1/2 heterodimers), TLR10 (receptor for undefined PAMPs) and TLR11 (receptor for uropathogenic bacteria and their products as well as *Toxoplasma gondii*) are cell surface receptors that primarily recognize bacterial structures. In contrast, TLR3 (receptor for double-stranded RNA and synthetic polysaccharides), TLR7 (receptor for single-stranded RNA), TLR8 (receptor for single-stranded RNA and imidazoquinolone, an antivirus drug) and TLR9 (receptor for unmethylated cytosine-phosphorothioate-guanine oligodeoxynucleotides (CpG-ODN)) are mainly localized intracellularly and recognize nucleic acids of microbial or viral origin. All TLRs can activate the nuclear factor-κB (NF-κB) signaling pathway, subsequently upregulating the expression of adhesion molecules and cytokines, and eventually leading to inflammatory responses [11,14,15].

**Figure 1:**
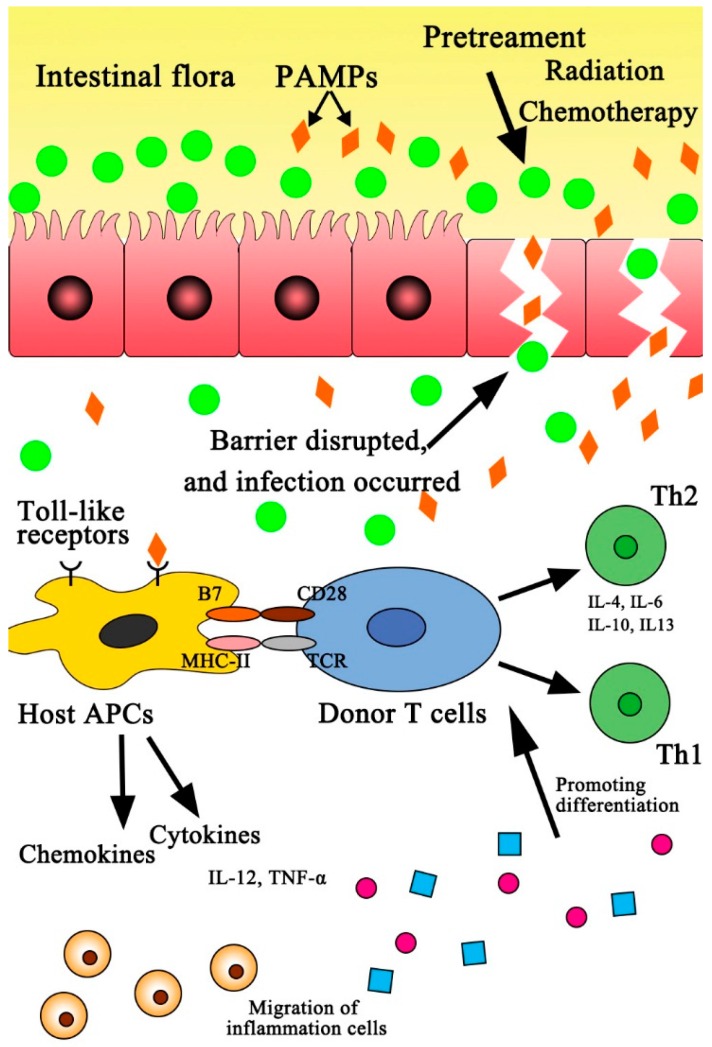
Schematic graph of GVHD initiated by intestinal flora. Intestinal flora enter the systemic circulation through damaged intestinal mucosa, and then interact with Toll-like receptors (TLRs), leading to the upregulation of major histocompatibility complex class II (MHCII) and costimulatory molecules B7 on host APCs. The upregulation of MHCII and costimulatory molecules B7 present the first and second activation signals, respectively, to donor T cells for the production of cytokines (including IL-1, IL-6, IL-12, IL-21, IL-22, IL-23, TNF-α and IFN-γ). The cytokines then induce the differentiation of a sub-population of donor T cells (Th1/Th2). Subsequently, the activated donor-derived T cells attack target tissues or organs (including intestine, liver and skin) by direct cytotoxicity, and eventually leading to GVHD. PAMPs = pathogen-associated molecular patterns; APCs = antigen-presenting cells; Th1 = helper T cell 1; Th2 = helper T cell 2.

The inflammatory cascades initiated by innate immune responses contribute to the occurrence of GVHD [7]. Besides, TLRs can intensify GVHD by subsequently inducing adaptive immune responses by recognition of exogenous microbial pathogens. In detail, TLRs expressed on recipient APCs can recognize and interact with the intestinal flora’s PAMPs, leading to the activation and maturation of APCs, which subsequently results in T cell migration or trafficking with an increasing production of inflammatory cytokines, including tumor necrosis factor-α (TNF-α), interleukin (IL)-1β, IL-6, IL-12 and interferon-γ (IFN-γ), which are all relevant to the activation of NF-κB signaling. In particular, IFN-γ can enhance antigen presentation by upregulating the expression of major histocompatibility complex (MHC) molecules on lymphoid and non-lymphoid tissues [16]. The activation of NF-κB signaling triggers the upregulation of MHC class II (MHCII) and costimulatory molecules B7, which present the first and second activation signals, respectively, to donor T lymphocytes for the production of cytokines (including IL-1, IL-6, IL-12, IL-21, IL-22, IL-23, TNF-α and IFN-γ). The cytokines then induce the differentiations of sub-populations of donor T cells, including helper T cell 1 (Th1), helper T cell 2 (Th2), helper T cell 17 (Th17) and regulatory T cell (Treg). Subsequently, allo-reactive donor-derived T cells attack target tissues or organs (including intestine, liver and skin) by direct cytotoxicity, eventually leading to GVHD (Figure 1). The current article reviews recent findings on the potential role of various TLRs in the occurrence and severity of GVHD, and explores new approaches for the prevention and therapies of GVHD.

## 2. TLR4 Signaling Pathway Activated by LPS

LPS, also known as lipoglycan and endotoxin, is microbial breakdown products during pre-transplant conditioning and has been considered as a driver of GVHD in experimental models [15,17]. TLR4 is broadly expressed by immune cells, such as monocytes, macrophagocytes, DC subsets, B cells and T cells, as well as by non-immune cells, such as human skin keratinocytes, embryo kidney cells, bronchial epithelia and intestinal epithelia [14,18]. In addition to recognition of LPS, TLR4 can also be activated by respiratory syncytial virus F protein, glycoinositolphospholipids (*Trypanosoma*), heat shock proteins and fibrinogen [19]. Since heat shock proteins may be released from dead or dying epithelial cells, in addition to impaired TLR4 signaling in response to PAMPs, there may also be delayed epithelial healing signaling in response to necrotic debris [17,20].

**Figure 2:**
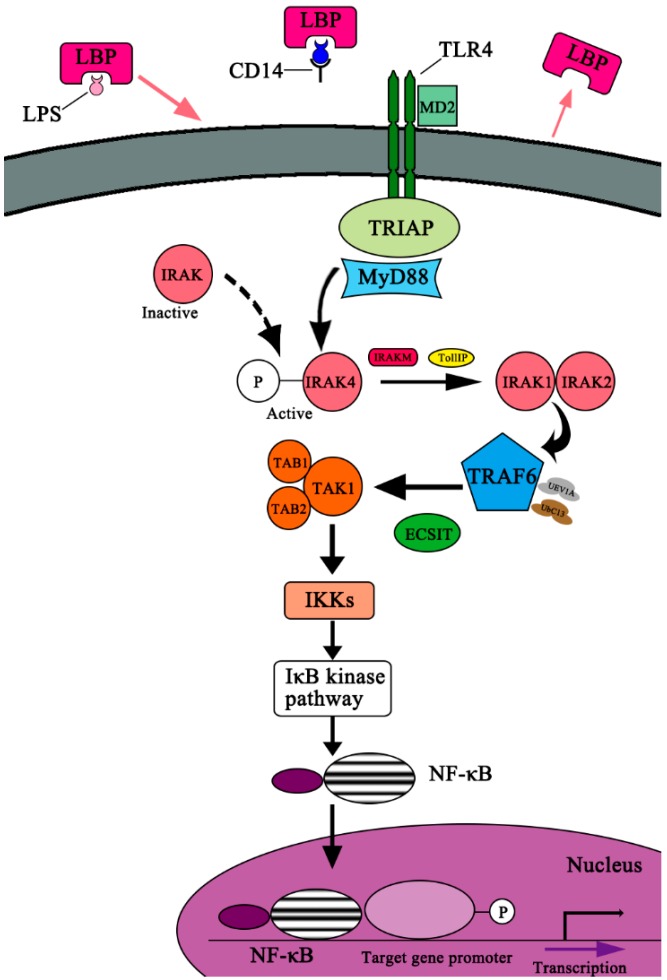
Diagram of LPS-induced TLR4 signaling. LPS, the breakdown bacterial component, binds to LBP, and then is released from the LPS-LBP complex, and is presented to CD14 and TLR4 on APCs, leading to the activation of TLR4. With the assistance of MD-2, an important component of activated TLR4, termed Toll/IL-1 receptor (TIR) homologous domain, binds to the C-terminus of cytoplasmic adaptor proteins MyD88, while the death domain (DD) at the N-terminus of MyD88 interacts with intercellular enzyme IL-1 receptor-associated kinase (IRAK), eventually resulting in the phosphorylation of IRAK and the activation of TNF-α receptor-associated factor 6 (TRAF-6). The phosphorylated IRAK binds to the activated TRAF, and the complex activates TGF-β-activated Kinase-1(TAK1), triggering the activation of inhibitor of κ polypeptide gene enhancer in B-cells (IκB) kinase, eventually activating the NF-κB signaling pathway. The NF-κB signaling pathway upregulates the expression levels of the target gene. The activated NF-κB signaling initiates the expression of target genes, leading to the damage of target organs. TLR4 = Toll-like Receptor-4; LBP = lipopolysaccharide-binding Protein; TIR = Toll-Interleukin-1-Receptor; TIRAP = Toll-Interleukin-1-Receptor Domain-containing Adapter Protein; MyD88 = Myeloid Differentiation Primary Response Protein-88; IRAK = Interleukin-1 Receptor-associated Kinase; IRAKM = Interleukin-1 Receptor-associated Kinase-M; TollIP = Toll-Interacting Protein; TRAF6 = Tumor Necrosis Factor Receptor-associated Factor-6; UbC13 = Ubiquitin-conjugating Enzyme-13; UEV1A = Ubiquitin-conjugating Enzyme E2-Variant-1; ECSIT = Evolutionarily Conserved Signaling Intermediate in Toll Pathways; TAK1 = TGF-β-activated Kinase-1; TAB1 = TAK1-binding Protein-1; TAB2 = TAK1-binding Protein-2; IKKs = Inhibitor of κ Light Polypeptide Gene Enhancer in B-Cells Kinase.

It has been demonstrated that intestinal permeability increases significantly in allo-HSCT patients who have received pre-transplant regimens such as chemotherapy and/or TBI [8]. Disruption of the gut-mucosa barrier perturbs the intestinal bacterial balance, with subsequent translocation of LPS and microorganisms from damaged intestinal mucosa into the circulation. In response to PAMPs, the LPS-mediated TLR4 pathway initiates inflammatory cascades and activates adaptive immune responses by upregulating the expression of costimulatory molecules on host APCs, including lipopolysaccharide-binding protein (LBP), cluster of differentiation 14 (CD14) and myeloid differentiation factor 2 (MD-2). The TLR4 signaling pathway depends on Myeloid Differentiation Primary Response Protein-88 (MyD-88), the downstream events of which are shown in Figure 2 [21,22,23]. Specifically, LPS, the breakdown bacterial component, binds to LBP which localizes on the surface of host APCs. LPS is then released from the LPS-LBP complex and is presented to CD14 and TLR4 on APCs, leading to the activation of TLR4. With the assistance of MD-2, an important component of activated TLR4 termed Toll/IL-1 receptor (TIR) homologous domain, it binds to the C-terminus of cytoplasmic adaptor protein MyD88, while the death domain (DD) at the N-terminus of MyD88 interacts with cytoplasmic enzyme IL-1 receptor-associated kinase (IRAK), eventually triggering the phosphorylation of IRAK and the activation of TNF-α receptor-associated factor 6 (TRAF-6). The phosphorylated IRAK binds to the activated TRAF-6, and then the complex activates TGF-β-activated Kinase-1 (TAK1), triggering the activation of inhibitor of κ polypeptide gene enhancer in B-Cells (IκB) kinase, eventually activating the NF-κB signaling pathway. The activated NF-κB signaling pathway can upregulate the expression levels of target genes. The upregulated MHCII and costimulatory factor B7 present the first and second activation signals, respectively, to donor T cells for the production of cytokines (including IL-1, IL-6, IL-12, IL-21, IL-22, IL-23, TNF-α and IFN-γ). These cytokines then induce the differentiation of sub-populations of donor T cells (Th1, Th2, Th17 and Treg). Subsequently, the activated donor-derived T cells attack target organs by direct cytotoxicity. The LPS-induced TLR4 signaling plays an essential role in the occurrence of GVHD [24,25]. It seems that LPS plays an essential role in the initiation of GVHD by means of TLR4 signaling, but there should be caution when jumping to the conclusion that LPS can affect the progression of GVHD in HSCT patients. Lorenz et al. [26] found that it is tended to have a reduced incidence of grade II to IV acute GVHD (aGVHD) in TLR-mutant patients compared with TLR4-intact patients (33.3% versus 47.2%), but they found that there were no statistically significant results. Elmaagacli et al. [27] demonstrated that in HSCT patients, a trend toward an increased risk of severe aGVHD was associated with *TLR4* gene mutations carried by both recipients and donors compared with recipients carrying the wild-type gene. However, the gene mutations are shown to have no effect on the transplant-related mortality, overall survival, and incidence of infectious complications. Due to the routine performance of bacterial gut decontamination in clinical HSCT patients, it may contribute, at least in part, to the observation of a strong effect of TLR4 signaling in mouse models and a weaker effect in human clinical studies.

There is growing evidence on the role of TLR4 in the pathogenesis of GVHD, ranging from gene polymorphisms to expression levels. Emerging data show that polymorphisms in the gene encoding TLR4 are relevant to the susceptibility of aGVHD; interestingly, two single-nucleotide polymorphisms (SNPs) of TLR4, Asp299Gly and Thr399Ile, are relevant to the enhanced immune responses and increased genetic risk for aGVHD, but the association between the severity of aGVHD and the SNPs was not statistically significant [27,28]. A much larger study population was needed to confirm the role of TLR4 in the pathogenesis of human GVHD. Furthermore, an increased risk for intestinal GVHD and severe GVHD was proved to be attributed by mutations of the *TLR4* (*Thr399Ile*) gene on both the patient and donor sides [27]. However, some data showed that mutations of TLR4 (Asp299Gly and Thr399Ile) in human leucocyte antigen (HLA)-matched sibling patients contributed to a reduced risk of GVHD, but an increased incidence for Gram-negative bacteremia tended to occur in HSCT patients with such TLR4 mutations [26]. Besides, LPS-induced TLR4 signaling plays a crucial role in the occurrence of chronic GVHD (cGVHD). Compared with non-GVHD patients after HSCT and healthy donor controls, TLR4-mediated NF-κB signaling-related genes including *TLR4*, *NF-κB*, *IL-6* and intercellular adhesion molecules 1 were significantly increased in patients with cutaneous cGVHD. The possible mechanism is the involvement of inflammation-mediated fibrosis in cutaneous cGVHD, which is mediated by TLR4-mediated NF-κB signaling [29].

On the other hand, the disruption of LPS-induced TLR4 signaling can ameliorate GVHD. The depletion of certain bacteria by treatment with an LPS inhibitor or an anti-endotoxin-neutralizing antibody, such as metronidazole and ciprofloxacin, can remarkably attenuate the detrimental influence on GVHD [30]. This can be explained by the fact that the elimination of LPS following anaerobic bacterial eradication has a negative effect on donor T lymphocyte activation via the TLR4 signaling pathway. In comparison with previous results showing that intestinal GVHD was initiated by TLR4 signaling pathways, a recent survey found that host TLR4 mutations could significantly increase the severity of GVHD, according to the survival and intestinal histopathologic changes in TLR4 mutant host mice [31]. It has been discovered that host TLR4 is critical for the induction of tissue-protective factors including cyclooxygenase-2 (COX-2) and COX-2-derived prostaglandin E2 (PGE2) and for protection against intestinal injuries during aGVHD [31,32]. This hypothesis may improve the strategies in terms of prophylaxis and therapy of aGVHD. Fukata et al. [18] demonstrated that it failed to upregulate COX-2 expression for TLR4-deficient mice in response to intestinal epithelial injury on account of the administration of dextran sodium sulfate. Their study also demonstrated that oral supplementation with PGE2 resulted in increased proliferation and decreased apoptosis of intestinal epithelia in TLR4-deficient mice. Thus, the failure of TLR4 signaling in host GVHD mice may result in the decreased expression of tissue repair factors, such as PGE2 and hepatocyte growth factor, in response to intestinal damage [32,33]. In summary, LPS-induced TLR4 signaling plays a dual role in the pathogenesis of GVHD in that host TLR4 can protect recipients against GVHD while donor TLR4 is essential to the occurrence and development of GVHD.

## 3. The TLR9 Signaling Pathway

As described above, TLR9 localizes within the endosomal compartment of immune cells and intestinal epithelial cells, and is activated by non-methylated CpG-ODN, which is derived from bacterial and viral DNAs such as malarial pigment hemozoin and herpes simplex virus DNA. The TLR9 signaling pathway has long been known to play a critical role in antitumor activity and immune responses; however, the underlying mechanism of the role of TLR9 in GVHD remains poorly understood. Since microbial breakdown products produced by conditioning regimens could include the TLR9 ligand CpG-ODN, the TLR9-mediated signaling pathway may play a critical role in the process of aGVHD [34]. In fact, the activation of the TLR9 signaling pathway promotes the transcriptional induction of genes that are important for host defense, producing Th1 which can promote chemokines and cytokine secretion including macrophage inflammatory protein-1, IFN-10, and other IFN-inducible proteins, thereby facilitating the regulation of antibody-dependent cell-mediated cytotoxicity induced by natural killer cells [16]. Similar to the LPS-induced TLR4 signaling pathway, the TLR9 transduction pathway also engages with the MyD88 adaptor protein.

The role of *TLR9* gene levels in the occurrence of GVHD has gained attention for many years. Emerging data showed that two SNPs in the human *TLR9* gene, T1486C and T1237C, have been confirmed to downregulate the expression of TLR9 mRNA [35]. Duramad et al. found that injection of immunoregulatory DNA sequences, which act systemically to block TLR-9 stimulation, into mice could prevent the systemic inflammatory response syndrome (SIRS) [36], suggesting that the occurrence of SIRS in allo-HSCT patients carrying the *TLR9* gene variant is significantly lower than that in patients with the wild-type *TLR9* gene. Therefore, it is reasonable to conceive that the *TLR9* gene variant can protect patients from CpG-ODN-induced lethal GVHD. However, based on an analysis of *TLR9* gene variants in 413 allogenic transplant patients and donors, Elmaagacli et al. [37] claimed that TLR9 SNPs do not affect the occurrence or severity of GVHD. Nonetheless, different results have been found in patients harboring a 1486C variant. A recent survey showed that compared to transplant patients with the wild-type gene, those with a *TLR9* gene variant at position 1486C had desirable outcomes, which was attributed to decreased transplant-associated mortality associated with GVHD [27]. Another study found that two tag SNPs at the donor side of the *TLR9* gene, t1174 A/G (rs352139) and t1635 C/T (rs352140), influenced the risk of developing aGVHD and cytomegalovirus reactivation [16]. A systematic review or meta-analysis is necessary to solve the dispute of whether the *TLR9* gene polymorphism can affect the occurrence or severity of GVHD.

In addition, increasing studies are performed to interpret the roles of key molecules involved in TLR9 signaling. It has been demonstrated that a single injection of the TLR9 agonist CpG-ODN greatly upregulated the production of TNF-α, IL-6 and IFN-γ, which was lethal in GVHD mice. In contrast, the blocking of TNF-α, but not IL-6 or IFN-γ, rescued GVHD mice from CpG-induced mortality [38]. Furthermore, the severity of aGVHD was remarkably reduced in TLR9-knockout host mice compared to the control group, with improved survival rates [39,40]. Experiments performed in bone marrow chimeric mice have revealed that TLR9 in the non-hematopoietic system has a significant influence on the outcome of GVHD models, whereas the presence or absence of TLR9 in hematopoietic cells has no effect on GVHD. This phenomenon is reasonable because non-hematopoietic cells, such as intestinal epithelia, express high levels of TLR9, and TLR9 directly participates in antigen presentation during GVHD [40,41]. In addition, the development of cGVHD appears to be associated with high levels of TLR9 expressed by B cells in increasing number, resulting in increased sensitivity to microbe-derived immunostimulatory CpG [42].

## 4. Other TLR Signaling Pathways

Studies on the understanding of other TLR signaling pathways remain scarce, let alone those on their roles in the pathogenesis of GVHD. Initially, researchers focused on the role of *TLR* gene polymorphisms. Based on the analysis of 305 HSCT patients, the genetic SNPs rs4833079 in TLR1, rs4837656 and rs17582214 in TLR4, rs10737416 in TLR5, rs6531656 in TLR6, and rs337629 in TLR10 were associated with the occurrence of aGVHD. Interestingly, two SNPs in the *TLR5* gene, rs2800230 and rs2800237, were associated with cGVHD [43]. In conclusion, it is reasonable to assume that these TLR signaling pathways are associated with the occurrence of GVHD. Since all TLRs can eventually initiate inflammatory responses by activating the NF-κB signaling pathway, with subsequent production of adhesion molecules and cytokines, it is thereby postulated that immune tolerance induction by TLR can make a difference in the occurrence and severity of GVHD. A new concept stating that frequent exposure to TLR stimulation may induce autoimmunity or tolerance, which is protective by limiting excessive inflammation, has gained attention. In vitro, repeated activation of TLRs induces unresponsiveness to the same TLR ligand in cell lines, B cells and plasmacytoid DCs. Repeated activation of TLR7 by low doses of TLR7 ligand-containing antigens can not only result in cross-tolerance, but also in enhanced responsiveness to other TLR ligands such as TLR2 and TLR9 [44]. Another study noted that following allo-HSCT, mice pretreated with 3M-011 (a TLR7/8 agonist) exhibited delayed GVHD and had significantly lower histological GVHD scores compared with those in the control group. The underlying mechanism is that IFN-γ produced by donor T cells leads to massive upregulation of indoleamine 2,3-dioxygenase (IDO) in host APCs, and IDO contributes to the reduced GVHD lethality [45]. Interestingly, GVHD inhibition was achieved by administering the TLR7/8 agonist before BM transplantation, in contrast to the GVHD acceleration with post-BM transplantation TLR agonist administration, with the latter likely caused by the release of overwhelming pro-inflammatory cytokines with inadequate control by IDO [46]. Thus, TLR7 signaling plays dual roles in local or systemic inflammatory responses. A TLR7 agonist can both induce the occurrence of GVHD and reduce the severity of GVHD. In addition, patients with GVHD have significantly increased expression of TLR5 mRNA, with the major TLR5 producers Lin-HLADR-CD33^+^CD16^+^ cells and CD14^+^CD16^−^ monocytes [47]. In conclusion, these data increase our understanding of the mechanism of different TLR signaling pathways in the occurrence and severity of GVHD, which can provide new insights for developing new clinically applicable therapeutic strategies to prevent GVHD in allo-HSCT patients.

## 5. Inhibition of TLR Signaling Pathways

The depletion of donor T lymphocytes or the inhibition of proliferative T lymphocytes can decrease the occurrence and severity of GVHD; however, there is a great risk of graft failure, a reduced graft-versus-leukemia (GVL) effect and an increased incidence of leukemic relapse, as well as an increased risk of severe infection. Therefore, an effective strategy for reducing GVHD without impairing the GVL effects or infection propensity is important. Emerging data showed that donor TLR4 and MyD88 deficiencies are protective against aGVHD [48], while activation of TLR9 with CpG-ODN in recipients markedly accelerates GVHD lethality [38,39]. Hence, novel therapeutic strategies that target TLR-mediated signaling pathways may be able to reduce the occurrence and severity of GVHD [49].

There is growing research in discovering new approaches relevant to TLR signaling pathways to intervene in the process of GVHD. LPS induces the release of massive inflammatory cytokines into the serum through the TLR4 signaling pathway with macrophage priming, thus leading to GVHD [50]. Conversely, antagonism of LPS (B975, a synthetic lipid-A analogue) significantly suppresses serum TNF-α levels and reduces both intestinal damage and systemic GVHD, without altering donor T cell activity toward host antigens in mice after experimental bone marrow transplantation [51]. In the meantime, the serum levels of LPS are relevant to the severity of GVHD in the target organs [45], while GVHD-associated lung injury can be significantly alleviated by treatment with an LPS antagonist [52]. However, the role of TLR4 on the donor side is distinct from that on the recipient side. A deficiency of activated TLR4 in donor hematopoietic cells prevents lung injury, whereas the presence or absence of TLR4 in recipient structural lung tissue has an insignificant effect on lung inflammation after transplantation. In conclusion, the signaling involved in lung injury thus may not be absolutely the same as that in the traditional TLR4 signaling pathway [52].

Heparan sulfate (HS), a ubiquitous component of the extracellular matrix, can activate TLR4 on DCs, leading to the enhancement of DC maturation and allo-reactive T cell responses [53]. Elevated serum HS levels aggravated GVHD both with regard to duration and severity of GVHD. In contrast, therapy with the serine protease inhibitor α1-antitrypsin was able to reduce the serum HS levels, leading to a reduction in donor allo-reactive T cell responses and GVHD severity. In conclusion, HS plays a crucial role in promoting GVHD, and the findings offer a new approach to preventing GVHD by lowering serum HS levels. Besides, Loiarro et al. synthesized the heptapeptide ST2825, which mimics the BB-loop of the MyD88-TIR domain, and found that ST2825 could block TLR signaling by interfering in MyD88 homodimerization [54]. In a co-immunoprecipitation assay, ST2825 inhibited the formation of the MyD88 dimerization by interacting with the TIR domains instead of the DD domains. ST2825 interfered with the recruitment of IRAK1 and IRAK4 by MyD88, attenuating the IL-1β-mediated activation of NF-κB. In addition, ST2825 suppressed the proliferation and differentiation of B lymphocytes in response to TLR9 stimulation. Thus, interference of MyD88 by ST2825 is a new approach to treating GVHD by inhibiting the recruitment of allo-reactive T lymphocytes activated by TLRs.

Interestingly, a study demonstrated that in experimental allo-HSCT models, the absence of MyD88 in donor T cells diminishes the GVL effect without attenuating the severity of GVHD [18], which may be contradictory to the fact that all TLRs, except TLR3, can induce GVHD by activating NF-κB signaling through the MyD88-dependent pathway. The activation of several types of adaptive immune responses as well as the generation of effectors including Th1, Th2, Th17 and Treg could be triggered by TLRs. However, cell-type-specific functions of MyD88 signaling remain poorly understood. In fact, in the donor/recipient strain combination (B6 → B6D2F1), donor CD4^+^ T cells are critical effectors of GVHD, while the GVL effect is dependent on the CD8^+^ T cells that can mediate cytotoxicity [55,56]. In the early phase after transplantation, recipients of MyD88-deficient T cells are found to have increased levels of Treg and Th2 cells and decreased levels of Th1 cells in the spleen and tumor-draining lymph nodes, but after the lapse of time, diminished Treg and Th2 cells are detected in recipients. This observation may be associated with reducing the GVL effect rather than GVHD-associated tissue damage. In vitro, however, the absence of MyD88 in CD8^+^ T cells results in defective cytotoxic activity against tumor tissue and a reduced secretion of pro-inflammatory cytokines to host antigens. In conclusion, the cause of maintaining the severity of GVHD with a reduced GVL effect is associated with functional dissociation of the two T cell subsets according to MyD88 deficiency in T cells. The dissociation of the GVL effect from GVHD may be promising in designing new effective therapeutic approaches against hematologic malignancies, which can harness the benefit of the GVL effect and reduce the toxicity of GVHD [18,35,57].

## 6. Conclusions

Innate immune responses which can be initiated by TLR signaling pathways significantly contribute to inflammatory cascades which lead to the recruitment of allo-reactive donor T cells to target organs, which is equivalent to GVHD. In detail, pre-transplant regimens including radiotherapy and/or chemotherapy lead to aggravated intestinal epithelial damage, with the subsequent dislocation of intestinal bacteria and their breakdown components including LPS, CpG-ODN and other ligands, resulting in the activation of the MyD88-dependent signaling pathway by ligation with TLR4, TLR9 or other TLRs. The consequent activated NF-κB signaling upregulates the expression of target genes to trigger the activation of donor T cells with the production of massive cytokines including IFN-γ, IL-1β, IL-6, IL-12 and TNF-α, leading to the recruitment of allo-reactive donor T cells to target organs. The blocking of any key molecules in TLR signaling pathways can inhibit the occurrence of GVHD, however, often accompanied by some undesirable complication such as reduced GVL and bacteremia. MyD88 in donor CD8^+^ T cells is proven to be critical for the preservation of GVL activity, regardless of the occurrence of GVHD, which may be a promising therapeutic target. In addition, protective factors in recipients such as COX2, PEG2 and IDO also contribute to reduced GVHD. Indeed, the eradication of intestinal bacteria with antibiotics such as metronidazole and ciprofloxacin has been clinically applied in HSCT patients. Further experiments and clinical trials are needed to interpret the role of TLRs in the induction of GVHD, a prerequisite to exploiting the use of specific TLR agonists or antagonists for treating GVHD.
